# Melatonin acts synergistically with auxin to promote lateral root development through fine tuning auxin transport in *Arabidopsis thaliana*

**DOI:** 10.1371/journal.pone.0221687

**Published:** 2019-08-28

**Authors:** Shuxin Ren, Laban Rutto, Dennis Katuuramu

**Affiliations:** Agriculture Research Station, Virginia State University, Petersburg, Virginia, United States of America; National Institutes for Quantum and Radiological Science and Technology, JAPAN

## Abstract

Melatonin (N-acetyl-5-methoxytryptamine) plays important roles in plant developmental growth, especially in root architecture. The similarity in both chemical structure and biosynthetic pathway suggests a potential linkage between melatonin and auxin signaling. However the molecular mechanism regulating this melatonin-mediated root architecture changes is not yet elucidated. In the present study, we re-analyzed previously conducted transcriptome data and identified 16 auxin-related genes whose expression patterns were altered by treatment with melatonin. Several of these genes encoding important auxin transporters or strongly affecting auxin transport were significantly down regulated. In wild type *Arabidopsis*, Melatonin inhibited both primary root growth and hypocotyl elongation, but enhanced lateral root development in a dose dependent manner. However, the lateral-root-promoting role of melatonin was abolished when each individual null mutant affecting auxin transport including *pin5*, *wag1*, *tt4* and *tt5*, was examined. Furthermore, melatonin acts synergistically with auxin to promote lateral root development in wild type *Arabidopsis*, but such synergistic effects were absent in knockout mutants of individual auxin transport related genes examined. These results strongly suggest that melatonin enhances lateral root development through regulation of auxin distribution via modulation of auxin transport. A working model is proposed to explain how melatonin and auxin act together to promote lateral root development. The present study deepens our understanding of the relationship between melatonin and auxin signaling in plant species.

## Introduction

Melatonin (N-acetyl-5-methoxytryptamine) was first discovered in the bovine pineal gland in 1958 [1] and then in plants about three decades later [2–3]. Melatonin has been extensively studied for its physiological roles since it was discovered. In animals, melatonin influences circadian rhythms, mood, sleep, food intake, seasonal reproduction, blood glucose, as well as the immune system [4–7]. In plants, melatonin influences photosynthesis and organ development [8–9], leaf senescence [10–11], as a defense against biotic and abiotic stresses [12–18] and root system architecture [19–21].

The chemical structure of melatonin is classified as an indolic compound and its biosynthesis in both animals and plants is through a tryptophan-dependent pathway [22–24]. Interestingly, the plant hormone auxin also belongs to the indole group in structure and at least partially through tryptophan-pathway biosynthesis [25]. The similarity in both chemical structure and biosynthetic pathway between melatonin and auxin suggests a potential linkage between melatonin and auxin signaling [11, 26]. Research to compare the physiological roles of melatonin and auxin was one of the major topics in melatonin studies since its discovery in plants, especially during the last decade [27]. Many studies strongly demonstrated that melatonin, like auxin, can promote growth in various plant species including economically important crops such as wheat, barley, rice, soybean, corn, tomato, pepper and cucumber [21, 28–32] and model species *Arabidopsis thaliana* [33]. When plants grow under stress conditions, melatonin plays an even more important role in promoting plant growth [34–38].

Root architecture is important for plant survival. Primary and lateral roots are major root architectural determinants and it is well known that auxin plays essential roles in lateral root development [39]. The effects of melatonin on root architecture have also been extensively studied. Arnao and Hernandez-Ruiz [28] demonstrated that melatonin treatment in lupin clearly affected the number and appearance of both adventitious and lateral roots. More recently, the effect of melatonin on adventitious and lateral root formation has also been demonstrated in other species such as tomato, cucumber, pomegranate and cherry [20–21, 40–43]. In the model species *Arabidopsis thaliana*, melatonin increased lateral root formation by up to 3-fold [19, 44]. Furthermore, the root growth promoting effect of melatonin has also been demonstrated by rice and *Arabidopsis* transgenic lines overproducing melatonin [45–46].

Although the physiological role of melatonin in lateral root development is well recognized, one of the major questions that remains unanswered is whether melatonin functions through auxin signaling. Using an auxin-inducible reporter DR5:GUS, Pelagio-Flores et al [19] demonstrated that melatonin action is independent of auxin signaling. However, other researchers have reported that can have both positive and negative effects on endogenous auxin production. For examples, treatments with exogenous melatonin have increase endogenous auxin level up to 7 folds [21, 47], while reduction in endogenous IAA levels have also been reported [48]. In addition, Wang et al. [33] reported that melatonin regulates the root meristem size through repression of auxin synthesis and transport. Furthermore, many auxin related transcription factors were up- or down- regulated following melatonin treatment in both rice and *Arabidopsis* [12, 49]. These studies suggest that melatonin may regulate root architecture by directly or indirectly modulating the auxin signaling pathway.

Previously, we conducted transcriptome analysis in *Arabidopsis* to understand how melatonin affects genome-wide gene expression in relation to plant defense systems [12]. In the present study, we re-analyzed the transcriptome data and identified 16 auxin related genes whose expression was altered upon melatonin treatment. Interestingly, several genes encoding important auxin transporters or those strongly affecting auxin transport were significantly down regulated. Real time qRT-PCR confirmed that all these transport-related genes were down regulated by melatonin, suggesting melatonin has a potential role in the regulation of auxin transport. In addition, melatonin inhibited both primary root growth and hypocotyl elongation, but enhanced lateral root development in a dose dependent manner in wild type *Arabidopsis*. However, the lateral-root-promoting role of melatonin was abolished when each individual null mutant affecting auxin transport including *PIN5*, *WAG1*, *TT4* and *TT5*, was examined. Furthermore, we discovered that melatonin synergistically acts with auxin to promote lateral root development in wild type, but such synergistic effects were absent in the knockout mutants of each individual auxin transport related gene examined. These results strongly suggest that melatonin enhances lateral root development through regulation of auxin distribution via modulation of auxin transport. The present study deepens our understanding of the relationship between melatonin and auxin signaling in plant species.

## Materials and methods

### Plant materials and growth conditions

*Arabidopsis* homozygous T-DNA knockout mutants of *pin5* (Salk_021738C and Salk_051354C), *wag1* (Salk_002056C and Salk_102906C) and *tir1* (Salk_151603C and Salk_090445C) in Columbia-0 (Col-0) background, and homozygous EMS mutants of *tt4* (CS85), *tt5* (CS86) and double mutant *tt4/tt5* in Landsberg (Ler-0) background were provided by *Arabidopsis* Stock Center at Ohio State University. All mutant lines together with Col-0 and Ler-0 were grown at 23°C with light intensity of 6950 Lux and a 14-h photoperiod to propagate enough seeds needed for the experiments. All T-DNA lines were PCR genotyped for their T-DNA status using combination of LBa1 primer with a pair of gene specific primers for the respected genes. For *tt4*, *tt5* and double mutant of *tt4/tt5*, transparent seed phenotype (after harvesting) was used to validate their homozygous status.

### Evaluation of melatonin effect on primary root and hypocotyl inhibition

To investigate how melatonin affects primary root and hypocotyl growth, seeds of Col-0 were surface sterilized in 50% bleach for 7.5 minutes and washed 5 times in sterile distilled water. The surface sterilized seeds were then directly germinated and grown vertically on half MS solid medium containing various amount of melatonin (10pM to 500μM) for 12 days prior to measuring the length of primary root and hypocotyl. Al seedlings were grown under 23°C with light intensity of 6950 Lux and a 14 hour photoperiod. The experiment was triplicated for statistical analysis.

### Evaluation of melatonin effect on lateral root development

Surface sterilized seeds were first germinated on half MS solid medium for 4 days and then the uniformed seedlings were transferred to the half MS medium containing 0, 50, 100, 200, 300, 400 and 500 μM melatonin and grown vertically for additional 6 days under 23°C with light intensity of 6950 Lux and a 14 hour photoperiod. Number of lateral roots were evaluated. The experiments were triplicated with at least 20 seedlings in each repeat setting. Based on the findings of this experiment, to simplify the rest of experiments, we used 100μM and 300μM melatonin for evaluation on lateral root development of all mutant lines.

To evaluate synergistic effect of melatonin with auxin, 4-day old Col-0 and Ler-0 seedlings were transferred to half MS medium containing 0, 100μm MT, 100pM 2,4-D and 100μm MT+ 100pM 2,4-D, and allow seedlings vertically grown for additional 6 days under same conditions of temperature, light intensity and photoperiod. The number of lateral root were then counted and recorded. All experiments were set for triplicate with 20 seedlings for each repeat.

Similarly, seeds of homozygous mutant lines of *pin5*, *wag1*, *tt4*, *tt5*, and double mutant *tt4/tt5* were also surface sterilized and germinated on half MS medium for 4 days. Lateral root development was investigated under same growing condition mentioned above on half MS medium containing 0, 100μM MT, 100pM 2,4-D, 100μM MT +100pM 2,4-D.

### RNA isolation and real-time qRT-PCR analysis

Three-week-old wild type Col-0 seedlings were removed from soil rinsed thoroughly, and then submerged in 300μM melatonin for 16 hours with gentle shaking. Mock solution was used as control. After melatonin treatment, total RNA was extracted using the QIAGEN RNeasy Mini Kit following the manufacturer’s instructions (QIAGEN). Prior to qRT-PCR, total RNA samples were treated with RNase free DNase to reduce DNA contamination. First strand cDNA was synthesized from one microgram total RNA using Superscript III reverse-transcriptase (Invitrogen) according to standard procedures provided with the kit. qRT-PCR was performed using SSoAdvanced SYBR Green Supermix (BioRad) on CFX-96 machine (BioRad) with the following parameters: 95°C for 3 minutes followed by 40 cycles of 95°C for 10 seconds and 60°C for 30 seconds. Gene expression was normalized via the Livak method using *Arabidopsis* Elongation Factor 1 (EF1, AT5G60390) as a reference gene [50]. All experiments were biologically triplicated and the primer pairs for all genes examined were listed in Table 1.

**Table 1:** Primer pairs of all mutant lines and control gene used in the study.

Gene Name	Forward Primer	Reverse Primer
ACS8	5’-CGGTTCTTCGTGCCATTGC -3’	5’-CTTCAATCTATCCAACGCTACC-3’
At3G12830	5’-CGAGATGGAGAGGTTCGTCG-3’	5’-CCATACTCTTGAGCAGATCGG-3’
ATGSTU1	5’-GCAGTGTACGAGAAGTTTGGAA-3’	5’-CAGGCAGGGCTTTAGCGAC-3’
GH3	5’-GCAGAGACGAAGACTATACCTG-3’	5’-GTTCAACGACTCCTCCATTTCC-3’
IAA3 (SHY2)	TCGGGCAAGATCTATGTTCA	ACCTTTTGCCCTGTTTCTGA
IAA17 (AXR3)	GGAGCACCGTACTTGAGGAA	TTTGCCCATGGTAAAAGAGC
LAX2	GTGAGCTAGTGCTGGGATG	GGCAAACATGGAGGAGAAGAAG
PIN5	CCATCATTCAGGCTGCTTTGC	CAACATCCCAAATATCACCGCTG
TT4	GGAGATAAAGCTAGGACTAAAGGA	CTAGTATGAAGAGAACGCACGC
TT5	GATCCTCTTCGCTCTCTCC	GGTGACACACCGTTCTTCC
WAG1	GGTTGAAGCCAAGGATTTGATAG	CATGCCTCTTGATATCTTGCG
EF1	GGTGACGCTGGTATGGTTAAG	GTCTGCCTCATGTCCCTAAC

### Statistical analysis

Data collected were subjected to statistical analysis using Graph Pad Prism 6. Differences among treatments were determined by one-way ANOVA followed by Duncan’s multiple range test. Data are presented as the mean ± standard deviation of three replicates. For qRT-PCR test, Student’s T-test was used to determine the significant changes in expression between control and melatonin-treated samples.

## Results

### Melatonin down regulates auxin transport-related gene expression

**Fig 1:**
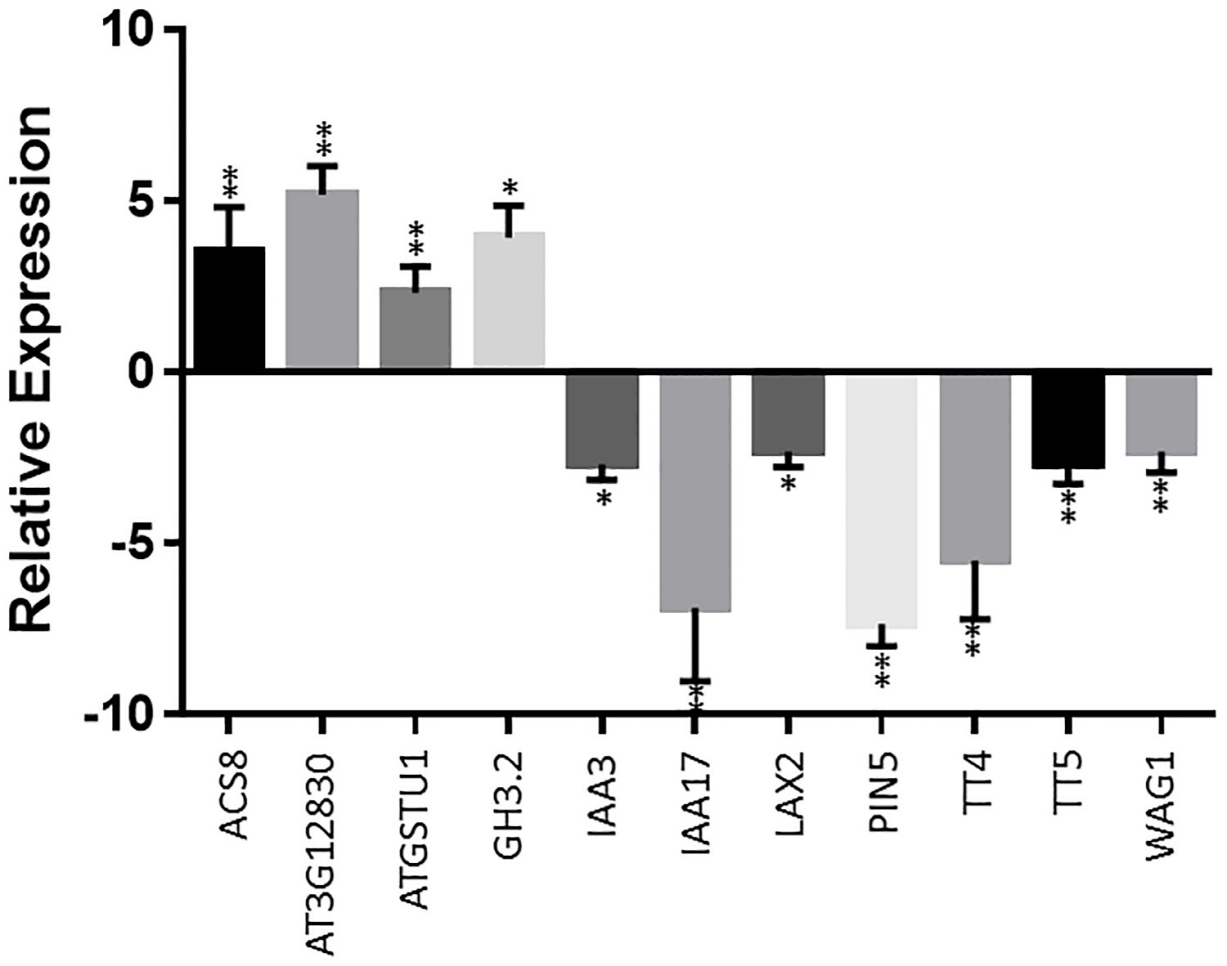
The expression pattern of auxin signal and auxin transport related genes altered by melatonin treatment. qRT-PCR was performed to assess the relative transcript levels of auxin related genes identified by RNA-seq. Samples were treated with or without 300μM melatonin for 16 hours. The transcript levels are expressed relative to that of *Arabidopsis* EF1 gene in each sample, and values are mean±SD (n = 4). Student t-test was used for P-value calculation. *P<0.05, **P<0.01.

Previously, transcriptome analysis in *Arabidopsis* revealed the role of melatonin in plant defense system [12]. Re-analyzing the RNA-seq data identified that the expression of 16 auxin related genes was significantly altered by melatonin treatment (Table 2). Of these, 12 were down regulated, with only 4 genes being up regulated by melatonin. Interestingly, five of the down regulated genes are involved in auxin transport, including *PIN5*, *TT4*, *TT5*, *WAG1*, and *LAX2*. Additionally, two Auxin/IAA proteins (*IAA3* and *IAA17*) were also down regulated by melatonin. To confirm the transcriptome data, qRT-PCR was conducted. Our previous research indicated that 300μM melatonin induces gene expression in a similar way as 1mM melatonin. Therefore, in our current study, we used 300μM melatonin to treat samples. Consistent with the transcriptome data, levels of *ACS8*, *GH3*.*3*, *AtGSTU1* and At3G12830 (*SAUR* like gene) expression were all increased in seedlings treated with melatonin. On the other hand, compared with the control, the expression of two IAA genes (*IAA3* and *IAA17*) and 5 auxin transport related genes was significantly decreased (Fig 1). These results strongly suggest that melatonin may function through regulation of intracellular auxin distribution.

**Table 2:** Auxin related genes with the expression affected by melatonin treatment by at least 2 fold in RNA-seq analysis.

Gene	Fold Change	Q-value	Annotation
ACS8	2.73046	0.000272	Encodes an auxin inducible ACC
At3G12830	2.17828	0.000875	SAUR-like auxin-responsive protein family
AtGSTU1	3.09797	0.000243	Encodes a member of the TAU glutathione S-transferase gene family and its expression in induced by exposure of auxin
GH3.3	2.2262	0.002972	Encodes an IAA-amino synthase that conjugates Asp and other amino acids to auxin
AXR3	-3.49394	0.001721	Transcription regulator acting as repressor of auxin-inducible gene expression
At1G29500	-3.84556	0.000914	SAUR-like auxin-responsive protein family
SAUR68	-3.52745	0.000905	Small auxin upregulated 68
TT5	-2.86673	0.000234	Catalyzes the conversion of chalcones into flavanones. Involved in response to auxin stimulus
At4G38860	-2.64102	0.000168	SAUR-like auxin-responsive protein family
At4G00880	-4.09193	0.000103	SAUR-like auxin-responsive protein family
TT4	-3.87023	8.77E-05	Encodes chalcone synthase (CHS) and involved in the regulation of auxin transport
PIN5	-4.37990	0.000123	A functional auxin transport. It localizes to endoplasmic reticulum (ER), mediating auxin flow from the cytosol to the lumen of the ER
At2G21050	-2.70401	0.000212	Encodes LAX2. A member of the LAX family of auxin influx carriers
CYP83A1	-3.3894	5.58E-06	Encodes a cytochrome p450 enzyme and has a role in auxin homeostasis
WAG1	-2.48533	0.000137	Encodes a protein-serine/threonine kinase that are nearly 70% identical to PsPK3 protein. Involved in auxin polar transport
SHY2	-3.70592	0.000198	SHY2/IAA3 regulates multiple auxin responses in roots

### Melatonin inhibits primary root growth and hypocotyl elongation in a dose-dependent manner

**Fig 2:**
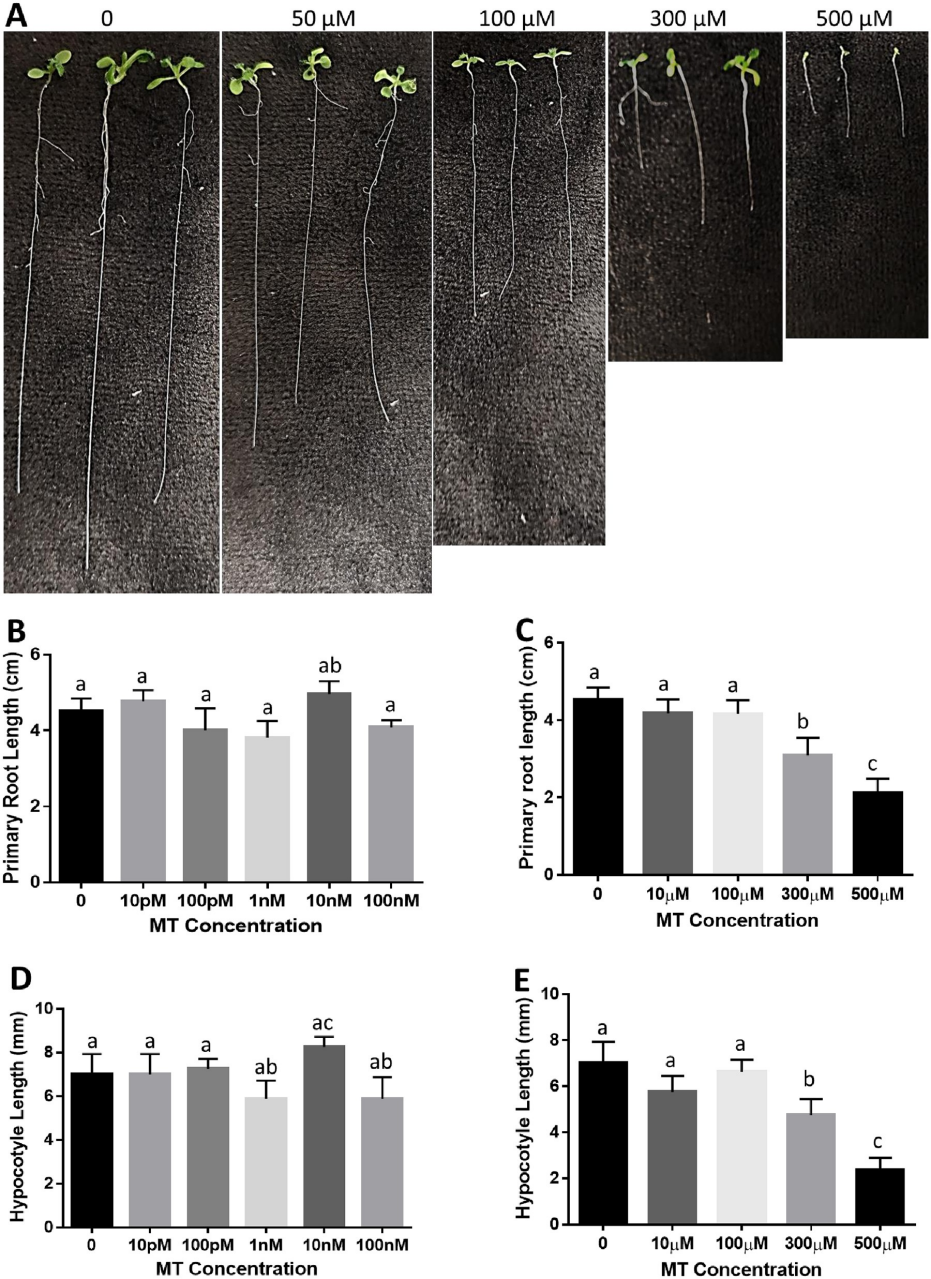
Effect of melatonin on *Arabidopsis* primary root length and hypocotyl elongation. Surface sterilized seeds of *Arabidopsis* ecotype Col-0 were directly germinated and grown on1/2 MS medium with different concentrations of melatonin for 10 days. Length of primary root and hypocotyl were measured. **(A)** Representatives of primary root length of wild type *Arabidopsis* seedlings treated with indicated concentrations of melatonin. Primary root length of *Arabidopsis* grown on medium with control and low (≤100nM) **(B)**, and high concentrations (≥10μM) **(C)** of melatonin. Hypocotyl elongation of *Arabidopsis* grown on medium with control and low (≤100nM) **(D)**, and high concentrations (≥10μM) **(E)** of melatonin. Three independent experiments were conducted for statistical analysis. Values are mean ±SD. Different letters indicate significant differences according to Duncan’s multiple range test (P<0.05).

To examine the effects of melatonin on primary root development in *Arabidopsis*, surface sterilized seeds of wild type Col-0 were germinated and grown vertically on half MS medium containing different amounts of melatonin for 12 days. We found that low concentrations of melatonin (10 pM to 100μM) did not promote or inhibit primary root growth. It was only when concentration increased to 300μM or above, that melatonin significantly decreased primary root growth (Fig 2A, 2B and 2C). These results are similar to those reported by Wang et al [33], but contradictory to findings by Pelagio-Flores et al [12] and Koyama et al [44]. We also investigated the effect of melatonin on hypocotyl elongation, and found that only high concentrations of melatonin significantly inhibited hypocotyl elongation (Fig 2D and 2E). Comparing the effect of auxin on root growth inhibition where much lower levels of auxin (less than 1 nM) are needed to inhibit primary root growth [51], our results, together with others (e.g. Reference 33), suggest that melatonin, unlike auxin, does not promote or inhibit primary root growth at physiological concentrations, but can inhibit primary root growth and hypocotyl elongation at much higher concentrations.

### Melatonin enhances lateral root development in a dose-dependent manner and acts synergistically with auxin

**Fig 3:**
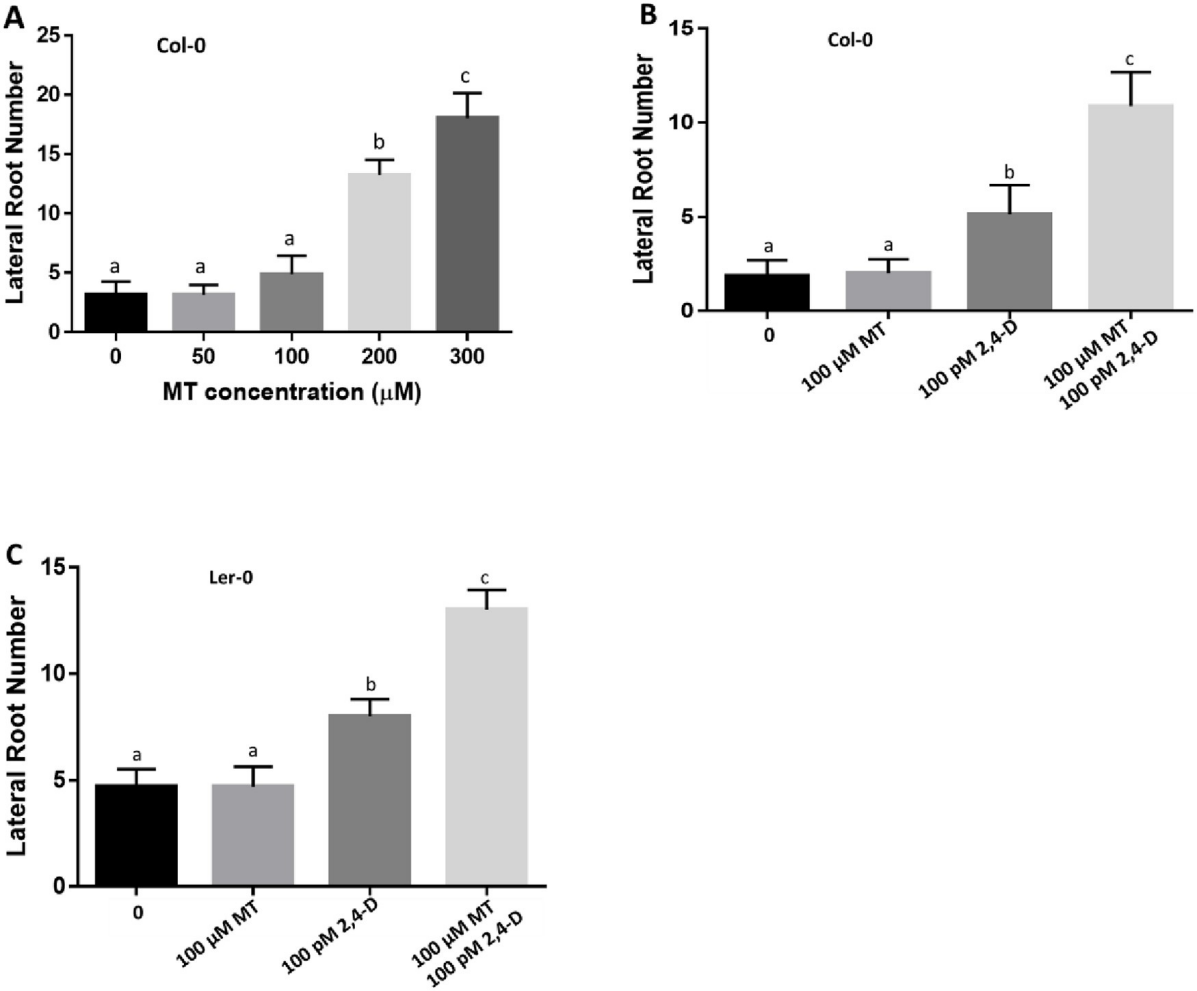
Synergistic effects of melatonin and auxin on lateral root development. A) Lateral root number of *Arabidopsis* ecotype Col-0 growing on ½ MS medium with control and increasing concentrations of melatonin. B) Effect of melatonin, auxin (2,4-D), and the combination on Arabidopsis Col-0 lateral root development. C) Effect of melatonin, auxin and the combination on *Arabidopsis* ecotype Ler-0 lateral root development. Three independent experiments with more than 15 seedlings per measure were conducted for statistical analysis. Values represent Mean ± SD, Different letters indicate significant differences according to Duncan’s multiple range test (P<0.05).

To investigate the role of melatonin on lateral root development, 4-day-old Col-0 seedlings were grown vertically on half MS medium with various concentrations of melatonin (50 to 500μM) for an additional 9 days. Again, low concentrations of melatonin (50 and 100μM) did not enhance or suppress lateral root development, but 200μM or higher melatonin content significantly promoted lateral root development in a dose dependent manner (Fig 3A). We also examined lateral root development at 500μM melatonin level, however, due to a dramatic reduction in primary root length (Fig 2A), lateral root development was also significantly affected and could not be counted. It is well known that auxin also plays an important role in promoting lateral root development [52]. However, whether there is a crosstalk between melatonin and auxin on lateral root development remains unknown. We therefore examined the interaction between melatonin and auxin on lateral root development. Shown in Fig 3B, 100μM melatonin did not increase the number of lateral roots, but as predicted, 100pM 2,4-D significantly increased the number of lateral roots. To our surprise, for 100μM melatonin in combination with 100pM 2,4-D, the number of lateral root growth is significantly increased. Similar results were also observed when using Ler-0 to test effects of melatonin, and combination of melatonin and auxin on lateral root development (Fig 3C). These results strongly suggest a synergistic effect between melatonin and auxin on lateral root development.

### Melatonin-mediated lateral root development and synergistic effect is abolished in null mutants of *pin5* and pin-related kinase *wag1*

**Fig 4:**
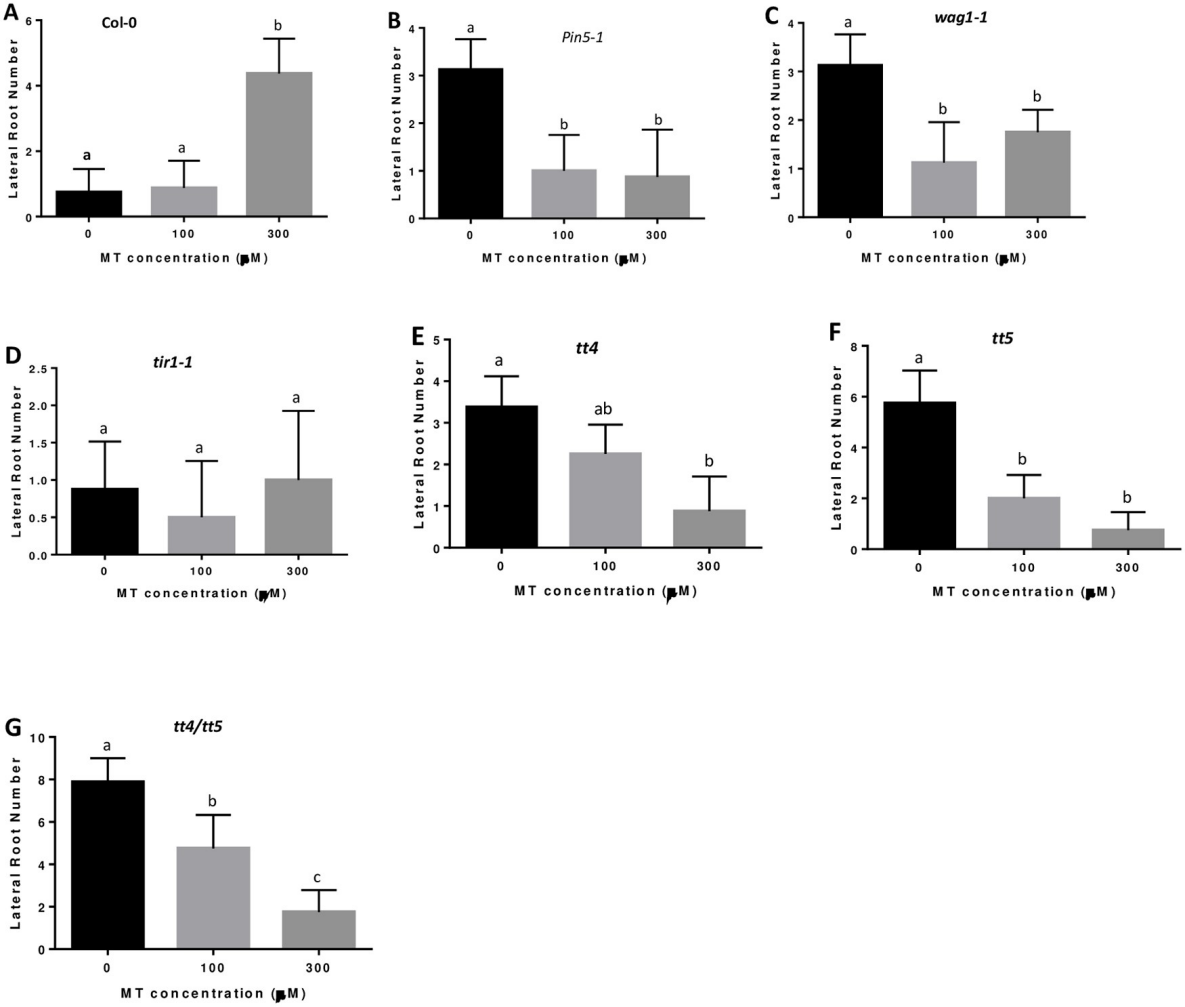
Auxin transport related mutants respond differently to melatonin on lateral root development. Lateral root number of *Arabidopsis* seedlings from wild type Col-0 (A), pin5-1 (B), tir1-1 (C), wag1-1 (D), tt4 (E), tt5 (F) and tt4/tt5 double mutant (G), growing on medium with control, 100μM and 300μM melatonin. Three independent experiments with more than 15 seedlings per measure were conducted for statistical analysis. Values represent Mean ± SD. Different letters indicate significant differences according to Duncan’s multiple range test (P<0.05).

**Fig 5:**
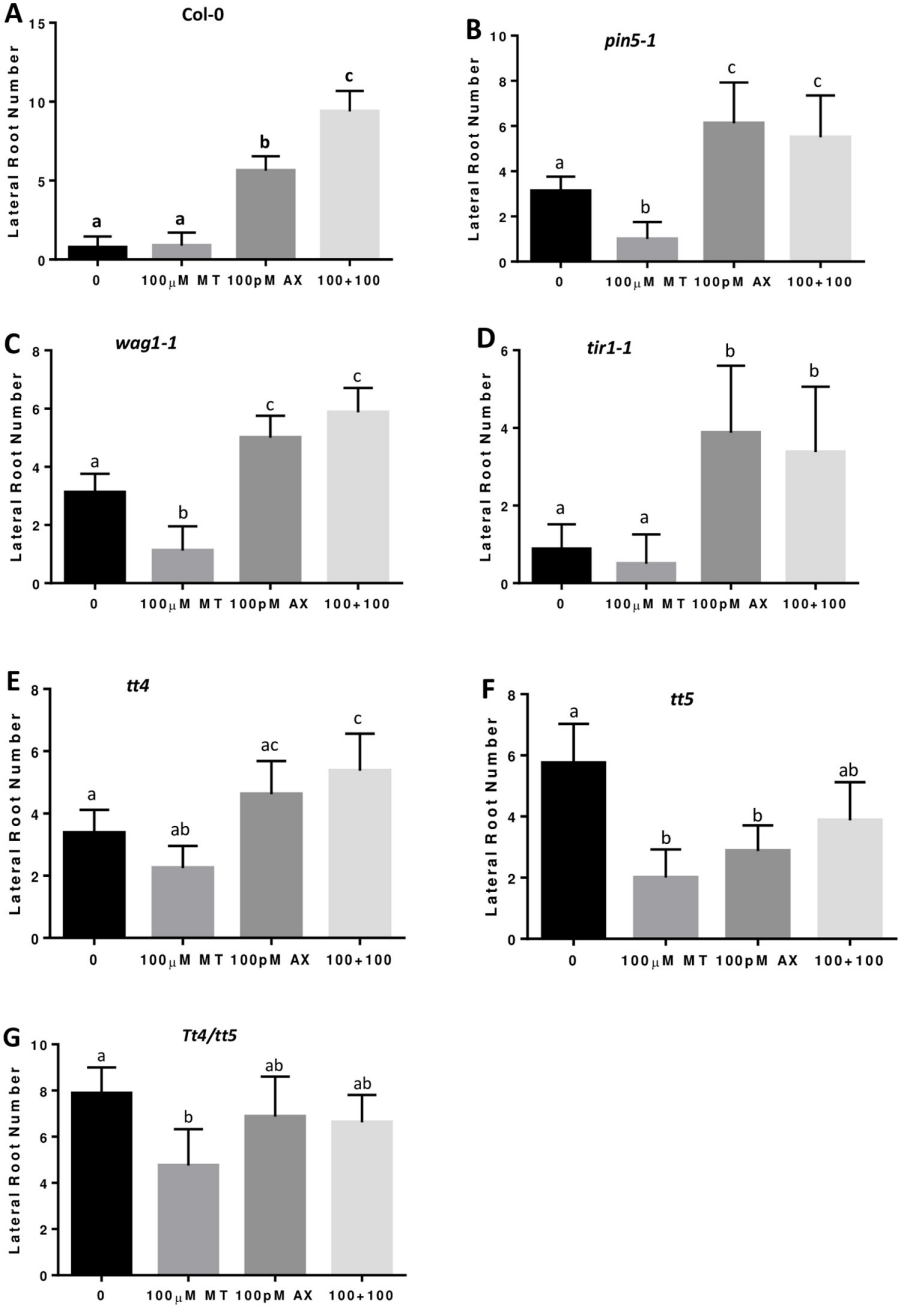
Synergistic effect of melatonin and auxin on lateral root development is abolished in auxin-transport-related mutants. Lateral root number of *Arabidopsis* seedlings from wild type Col-0 (A), pin5-1 (B), wag1-1 (C), tir1-1 (D), tt4 (E), tt5 (F) and tt4/tt5 double mutant (G), growing on medium with control, 100μM melatonin, 100pM 2,4-D and the combination. Three independent experiments with more than 15 seedlings per measure were conducted for statistical analysis. Values represent Mean ± SD. Different letters indicate significant differences according to Duncan’s multiple range test (P<0.05).

Given that melatonin down regulates both *PIN5* and *WAG1* (Fig 1), we hypothesize that melatonin-mediated lateral root development is through control of auxin distribution. To test this hypothesis, lateral root development was examined for both homozygous T-DNA mutants of *PIN5* (*pin5-1*) and *WAG1* (*wag1-1*). Both *pin5-1* and *wag1-1* mutants developed more lateral roots than the wild type on half MS medium (Fig 4A, 4B and 4C), most likely due to the dysfunction of the internal auxin relocation. However, unlike that of Col-0, the number of lateral roots did not increase for both *pin5-1* and *wag1-1* mutants with the addition of melatonin. Instead, lateral root development was significantly reduced by melatonin in both *pin5-1* and *wag1-1* mutants (Fig 4B and 4C). Exogenous auxin increased lateral root development on both *pin5-1* and *wag1-1*, however, with the combination of melatonin and auxin, the synergistic effects on lateral root development were abolished in *pin5-1* and *wag1-1* mutants (Fig 5B and 5C). To confirm these observations, we further confirmed these results with a different set of *pin5* and *wag1* T-DNA knockout mutants (*pin5-2* and *wag1-2* respectively). Our results strongly suggest that melatonin regulates lateral root development through control of auxin relocation within cells.

Unlike other PIN proteins, that are located on either the cell or nuclear membrane (in case of PIN8) and function as auxin efflux carriers to transport auxin out of the cell or nucleus [53], PIN5 and PIN6 are located in the endoplasmic reticulum (ER) and may serve as influx carriers to transport auxin from the cytosol to nucleus [54]. TIR1 is an auxin receptor located in the nucleus and its function relies on PIN5 to transport auxin into nucleus. Therefore we predict that *TIR1* null mutant should behave similar to *pin5* even though melatonin did not down regulate *TIR1* expression. To test this possibility, we examined the lateral root development for two independent T-DNA knockout lines of *TIR1* gene. The results showed that even 300μM melatonin did not increase lateral root development for *tir1* mutants (Fig 4D). However, 100pM 2,4-D did increase lateral root development for *tir1* mutant, but with significantly less strength than that of the wild type (Fig 5A and 5D). Furthermore, synergistic effect between melatonin and auxin was also not observed in *tir1* mutants (Fig 5D).

### Melatonin inhibits lateral root development in single mutant of *tt4*, *tt5* and their double mutant

*Arabidopsis TT4* encodes a chalcone synthase and *TT5* catalyzes a conversion of chalcones into flavonones [55–56]. Numerous reports demonstrate that mutations on the flavonoid pathway in both *Arabidopsis* and tomato play important roles on lateral root development by influencing auxin transport [57–60]. However, proposed mechanisms by which flavonoids affect lateral root growth are contradictory. For example, Brown et al [57] reported that a *tt4* mutant had increased lateral root development, while Buer and Djordjevic [61] demonstrated that *tt4* had fewer lateral roots than the wild type. Since melatonin treatment significantly reduced the expression of both *tt4* and *tt5* (Table 1; Fig 1), we examined the effects of melatonin on lateral root development in both *tt4* and *tt5* single mutants and the *tt4/tt5* double mutant. Consistent with Brown et al [57], both *tt4* and *tt5* as well as the *tt4/tt5* double mutant developed significantly more lateral roots on half MS medium (Fig 4). The number of lateral roots in *tt4* was moderately increased in comparison to the wild type. However, both the *tt5* single mutant and the tt4/tt5 double mutant showed a dramatic increase in lateral root development on half MS medium (Fig 4). We also examined lateral root development of *tt4*, *tt5* and the *tt4/tt5* double mutant on half MS medium with the addition of 300μM melatonin. As shown in Fig 4E, 4F and 4G, addition of 300μM melatonin led to a significant reduction in the number of lateral roots in *tt4*, *tt5* and *tt4/tt5* mutants compared to that of wild type Columbia (Fig 4A).

To further examine the relationship among melatonin, auxin and flavonoid pathway, we compared the synergistic effects of melatonin and auxin between wild type Columbia and *tt4*, *tt5* mutants. As shown in Figs 3B and 5A, 100pM 2.4-D enhanced lateral root development and 100μM melatonin and 100pM 2,4-D synergistically increased lateral root formation in the wild type. However, 100pM 2,4-D did not significantly promote lateral root development in *tt4* and *tt4/tt5* mutants (Fig 5E and 5G), and even reduced lateral root development in the *tt5* mutant (Fig 5G). Furthermore, the synergistic effects observed in the wild type were abolished in both *tt4*, *tt5* single mutants and the *tt4/tt5* double mutant (Fig 5E, 5F and 5G).

These results, together with the report on flavonoid mediated auxin transport [57], strongly demonstrate that crosstalk among melatonin, the flavonoid pathway, and auxin transport plays a key role in lateral root development in *Arabidopsis thaliana*.

## Discussion

Melatonin exists in all plant species so far examined. Since it was identified in plants in 1995 [2–3], considerable studies, especially during the last decade, have suggested that melatonin is an important regulator in controlling root development [19, 28, 33, 40, 44–45, 49, 62]. Due to similarity in structure and a common precursor (Tryptophan) in their biosynthesis, research on the relationship between melatonin and auxin has drawn much attention. Among its physiological roles, melatonin has been repeatedly demonstrated to have auxin-like actions [11, 26]. However, molecular studies on whether or not melatonin acts independently of auxin signal are not conclusive. For example, Pelagio-Flores et al [19] and Koyama et al [44] demonstrated that melatonin acts independently of auxin signaling, while others approved that melatonin acts by modulating auxin response [21, 33, 49]. In the present study, low concentrations of melatonin (10pM to 100μM) did not alter primary root growth, but significant inhibition was observed when melatonin concentrations were raised to 300μM or higher (Fig 2). This result is consistent with the report by Wang et al [33], but different from Pelagio-Flores et al [19], where even 600μM melatonin did not affect primary root growth. When testing the role of melatonin on lateral root formation, we also found that low concentrations of melatonin did not affect lateral root development while high concentrations (200μM to 300μM) drastically increased lateral root development (Fig 3). A positive effect on lateral root development by exogenous melatonin is widely reported but effective concentrations vary [28–29]. Our current results, together with others, suggest that melatonin may have a similar function to auxin in regulating root development, but concentrations needed for melatonin are much higher than that of auxin to reach similar levels of promotion or inhibition of root development. With the recent discovery of a potential melatonin receptor in *Arabidopsis thaliana* [63], the door opens for melatonin to be considered as a new plant hormone. However, as Arnao and Hernandez-Ruiz [64] suggested that melatonin is so diverse in its actions and would be more appropriate to be defined as a plant master regulator.

Most of studies conducted so far were focused on melatonin’s physiological role and examined its auxin-like function in regulating plant growth and development. However, works on evaluating the interactions between melatonin and auxin have drawn less attention. To date, we only identified two reports that investigated the effect of a combination of melatonin and auxin on 1) adventitious root regeneration in cherry rootstocks [42] and 2) root meristem size in *Arabidopsis* [33]. In the first report, the authors did not conclude the synergistic effect of melatonin and auxin on adventitious root development, but the results did indicate some interactions between the two molecules. For example, treatment with both 0.1μM melatonin and 4.92μM IBA significantly increased the number of adventitious roots generated when compared with treatments with same concentrations of melatonin and applied separately with IBA, effects varying according to genotypes [42]. In the second report, Wang et al [33] also indicated that the simultaneous presence of 100nM IAA and 600μM melatonin led to more severe decrease in root meristem size than 600μM melatonin alone. In our current study, we examined the effect of a combination of melatonin and auxin on lateral root development in *Arabidopsis*, and found that melatonin acts synergistically with auxin to control lateral root development in *Arabidopsis* ecotype Col-0 (Fig 3B). However, in ecotype Ler-0, we found that a combination of melatonin and auxin could also have an additive effect. Such genetic effects: synergistic, additive, or both, may play important roles in regulating lateral root development. Auxins are known to be an essential plant hormone involved in control of root development [65–68], while recent studies demonstrated that melatonin also plays an important role in plant lateral root development [19, 44, 49]. However, the combined effect of these two molecules on lateral root development remains unknown. Our current finding of synergism or additive effect between melatonin and auxin broadens our understanding of the relationship between melatonin and auxin and warrants further study on the molecular mechanisms regulating this synergistic action.

Lateral root formation is closely regulated by auxin signaling. Many mutations involved in auxin signaling, for examples, *iaa1*, *iis3*, *iaa14*, *iaa19* and *iaa28* [69–74], altered plant capacity for lateral root development. In studying the mechanisms of melatonin-mediated lateral root development, Liang et al [49] identified at least 6 IAA genes in rice that were up regulated by melatonin. On the contrary, our previous transcriptome analysis in *Arabidopsis* did not find any up regulated IAA genes [12], but instead, found two IAA genes (*iaa3* and *iaa17*) that were significantly down regulated by melatonin. Such discrepancy may be caused by different concentrations used to treat the materials or alternatively, maybe due to different mechanisms of melatonin-mediated lateral root development between species. Nevertheless, 5 genes encoding either auxin transporters or protein enzymes involved in regulating auxin transport were dramatically down regulated by melatonin in our *Arabidopsis* gene expression analysis (Table 1 and Fig 1). Such significant down-regulation of auxin-transport-related genes by melatonin strongly suggests that melatonin-mediated lateral root development is closely associated with a fine tuning of auxin partitioning within the cells through control of auxin transport processes in *Arabidopsis*. In consistence, we observed that the melatonin-mediated lateral root development phenotype was abolished in homozygous null mutants of *pin5*, *wag1*, *tt4*, and *tt5* and the *tt4/tt5* double mutant (Fig 4). Additionally, the synergistic effect of melatonin and auxin on lateral root development was also abolished in these null mutants (Fig 5). These results further support the hypothesis that melatonin regulates lateral root development via modulation of auxin partitioning in cells.

Different from other PIN proteins, that are localized in the plasma membrane, and function to mediate directional auxin fluxes among tissues [53], PIN5 is located in the ER and mediates intracellular auxin partitioning and homeostasis [54]. Although it is believed that auxin, as a small molecule, can enter the nucleus through passive diffusion without restriction (cytosol to nucleus diffusion) [75], recent studies demonstrate that the nuclear uptake of auxin is driven by processes other than diffusion and ER to nucleus flux dominates over the diffusion [76]. Since PIN5 is located in the ER, it is probable that it plays a rate-limiting role in regulating nuclear uptake of auxin [76].

The functions of PIN proteins can be regulated by multiple factors. For example, auxin itself can up-regulate the transcription of many PIN genes, however, only the *PIN5* gene is down-regulated by auxin [54]. In addition, PIN’s function is also linked to their phosphorylation status [77]. For example, the protein kinase *PINOID* and its homologs *WAG1* and *WAG2* play important roles in phosphorylation of PIN proteins [78–79]. Interestingly, our results show that both *PIN5* and its potential kinase *WAG1* were down-regulated by treatment with melatonin (Fig 1). The effect of melatonin on lateral root development was abolished in both null mutants of *pin5* and *wag1* (Fig 4), and the synergistic effect observed in the wild type control also abolished in both null mutants (Fig 5). These results suggest that melatonin regulates lateral root development through control of nuclear auxin uptake via *PIN5*-mediated influx (ER to nucleus) channel.

The *PIN* genes’ activity can also be regulated by endogenous flavonoid regulators, although the mechanism behind this action is not yet understood [80]. The enzymes involved in flavonoid synthesis such as *TT4* and *TT5* also affected long distance auxin transport and altered lateral root development capacity when mutation occurred on either *TT4* or *TT5* [57, 59]. In the *Arabidopsis tt4* mutant, the rate of auxin transport was significantly increased [57–59], however, the flavonoids interact with regulatory proteins rather than directly with the PIN auxin efflux carriers [81–83]. Consistent with these discoveries in *Arabidopsis*, Wasson et al [84] also demonstrated that *PIN* family gene transcriptions were not significantly changed in the generated flavonoid deficiency mutant in *Medicago truncatula*. In the present study, we found that melatonin significantly reduced the expression of both *tt4* and *tt5* in *Arabidopsis* (Fig 1). Null mutants of *tt4* and *tt5* as well as the double mutant *tt4/tt5* generated more lateral roots on half MS medium (Fig 4). However, with the addition of melatonin, the number of lateral roots was dramatically reduced and synergistic action between melatonin and auxin was also abolished (Figs 4 and 5).

**Fig 6:**
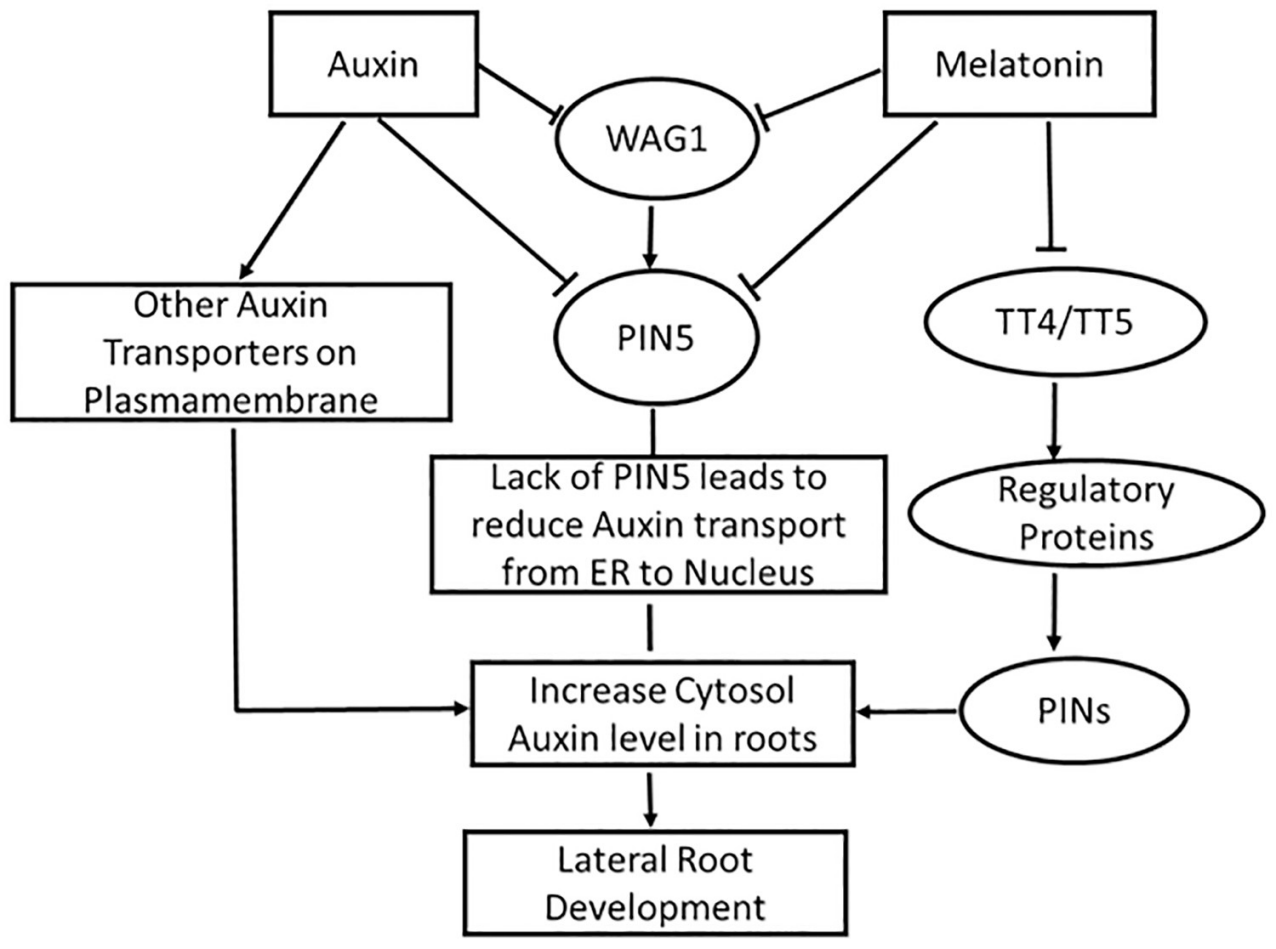
Working model describing the mechanism of auxin and melatonin in regulating lateral root development.

Based on our results from this research, together with other reports, we propose a working model describing how auxin and melatonin interact to regulate lateral root development (Fig 6). On the one hand, exogenous auxin activates many auxin transporters located in the plasma membrane and leads to ample flow of auxin into root cells. In the meantime, the exogenous auxin down-regulates the *PIN5* influx carrier, either directly or through *WAG1* protein kinase [85], affecting auxin transport from ER to nucleus [54] within the cell. The direct cause of exogenous auxin is to increase free auxin levels in the cytosol and stimulate lateral root development. On the other hand, by down regulating the *PIN5* either directly or indirectly through regulatory protein kinase *WAG1*, exogenous melatonin prevents auxin transport from cytosol to ER to nucleus. Furthermore, exogenous melatonin also down regulates flavonoid biosynthesis by reducing transcripts of *TT4* and *TT5*. Down regulation of flavonoids directly affects regulatory protein activity and hence indirectly activates auxin transporters in the plasma membrane. These dual actions by exogenous melatonin lead to a high level of auxin in the cytosol and result in increased lateral root formation. Currently it is not known how this cytosolic auxin works to regulate lateral root formation, but one possibility is that cytosolic auxin elevates the cytosolic Ca^2+^ ion levels and hence calcium signaling will eventually lead to control of lateral root development [86]. It is also worth mentioning that even though *PIN5* is down-regulated by both auxin and melatonin, it does not mean that auxin cannot be up taken into the nucleus. At least some auxin can still get into the nucleus through diffusion or by other potential influx transporters located on ER, such as PIN6 or newly identified PIN-LIKE (PILS) proteins [54, 87]. Interestingly, the original site of melatonin synthesis has been shown to be in the mitochondria in both animal and plant species [88–89], and more specifically to be on the matrix of mitochondria in animals [90]. Such an arrangement would make it easy to release melatonin into the cytosol and for it to interact with auxin to control lateral root development. Additional investigation is needed to further refine this working model. It is difficult to measure auxin fluxes within the cells, however, with recently developed tools and mathematic models [76], together with other techniques, this type of measurement may be feasible in the near future.
